# N-[3-(Aminomethyl)benzyl]acetamidine (1400 W) as a Potential Immunomodulatory Agent

**DOI:** 10.1155/2014/491214

**Published:** 2014-06-05

**Authors:** Anna Mertas, Hanna Duliban, Ewelina Szliszka, Agnieszka Machorowska-Pieniążek, Wojciech Król

**Affiliations:** ^1^Department of Microbiology and Immunology, Medical University of Silesia in Katowice, Jordana 19, 41 808 Zabrze, Poland; ^2^Regional Centre of Blood Donation and Blood Treatment in Opole, Kośnego 55, 45 372 Opole, Poland; ^3^Department of Orthodontics, Medical University of Silesia in Katowice, Plac Traugutta 2, 41 800 Zabrze, Poland

## Abstract

This study was designed to investigate the relationship between NO, IL-12, and TNF-**α** production by J774A.1 macrophages activated with LPS and IFN-**γ** in the presence of N-[3-(aminomethyl)benzyl]acetamidine (1400 W). 1400 W is a novel, highly selective inhibitor of inducible nitric oxide synthase (iNOS). We compared the obtained data with the effect of N^G^-monomethyl-L-arginine (L-NMMA) (a nonselective NOS inhibitor) and L-N^G^-(1-iminoethyl)lysine (L-NIL) (a relatively selective inhibitor of iNOS activity) on cells in this model. To investigate the involvement of an exogenous NO on IL-12 and TNF-**α** production we used NO donor—S-nitrosocaptopril (S-NO-Cap). The most potent inhibitor of NO generation was 1400 W. This compound also markedly increased IL-12 p40 secretion and decreased TNF-**α** release. L-NIL suppressed both NO and TNF-**α** production, but it did not change IL-12 p40 synthesis. The effect of L-NMMA on NO generation was weaker than other inhibitors. Moreover, it decreased TNF-**α** secretion slightly but not significantly. IL-12 p40 production by stimulated cells was inhibited by S-NO-Cap in a dose dependent manner, but no effect on TNF-**α** release was observed. The potency and selectivity of 1400 W as an inhibitor of iNOS and cytokine release modifier are encouraging for therapeutic use.

## 1. Introduction

Cytokines are low molecular weight polypeptides that initiate the inflammatory response and define the magnitude and the nature of the acquired immune response. Interleukin 12 (IL-12), tumor necrosis factor alpha (TNF-*α*), and interferon gamma (IFN-*γ*) are three inflammatory mediators, which have significant impact on the cytokine balance and type of immune response. Additionally, nitric oxide (NO) seems to participate in this regulation [1, 2]. IL-12 (a heterodimer composed of two subunits: p35 and p40) induces commitment from the T helper 0 (Th0) to Th1 phenotype [3, 4]. NO has been suggested to inhibit IL-12 transcription and to act as negative feedback on Th1 cell development [5]. Also TNF-*α* seems to be a specific inhibitor of IL-12 p40 secretion from human macrophages [6].

IL-12, TNF-*α*, and NO are produced and released by macrophages upon activation by a variety of immunological stimuli, such as lipopolysaccharide (LPS) and cytokines. NO is synthesized from L-arginine by the enzyme—NO synthase (NOS), which is either constitutive (endothelial—eNOS and neuronal—nNOS) or induced (iNOS) by bacterial products and cytokines [7, 8]. High output NO production from activated macrophages is a result of iNOS expression [9, 10]. There are known several NOS inhibitors; most of them are analogs of the substrate L-arginine [11]. Preservation of physiologically important NOS functions might require use of isoform-selective inhibitors. N-[3-(aminomethyl)benzyl]acetamidine (1400 W) is a slow, tight binding, and highly selective inhibitor of iNOS [12, 13].

The purpose of this study was to investigate the relationship between NO, IL-12, and TNF-*α* production by J774A.1 macrophages activated with LPS and IFN-*γ* in the presence of 1400 W. We compared obtained data with the effect of N^G^-monomethyl-L-arginine (L-NMMA) (a nonselective NOS inhibitor) and L-N^G^-(1-iminoethyl)lysine (L-NIL) (a relatively selective inhibitor of iNOS activity) on cells in this model. To investigate the involvement of an exogenous NO on IL-12 and TNF-*α* production we used NO donor—S-nitrosocaptopril (S-NO-Cap).

The J774A.1 cell line was used in our study because this kind of cells is a widely used useful model to study the process of nitric oxide (NO) synthesis. In J774A.1 murine monocyte-macrophage cell line NO production significantly increases in the presence of LPS and IFN-*γ*, or LPS alone [14–17].

## 2. Materials and Methods

### 2.1. Reagents

1400 W, L-NIL, L-NMMA, and S-NO-Cap were purchased from Calbiochem-Novabiochem Corporation (La Jolla, CA, USA). LPS from* Escherichia coli* serotype O127:B8 and trypan blue were purchased from Sigma Chemical Company (St. Louis, MO, USA). Recombinant mouse IFN-*γ* was obtained from Genzyme Corporation (Cambridge, MA, USA).

### 2.2. Cell Culture

The mouse macrophage cell line J774A.1 was obtained from Deutsche Sammlung von Mikroorganismen und Zellkulturen (Braunschweig, Germany). Cells were maintained in an atmosphere of 5% CO_2_, at 37°C in RPMI 1640 medium (BioWhittaker, Walkersville, MD, USA) supplemented with 10% fetal bovine serum, 2 mM glutamine, 100 U/mL penicillin, and 100 *μ*g/mL streptomycin (Gibco BRL Life Technologies, Paisley, UK). The cells were cultured in 50 cm^2^ plastic flasks (Nunc A/S, Roskilde, Denmark). For experiments cells were detached by vigorous pipetting and, after centrifugation, suspended in fresh medium. Macrophages at a density of 1 × 10^6^ cells/mL were activated with a combination of LPS (100 ng/mL) and IFN-*γ* (25 U/mL) for 18 h. Incubations were performed in 24-well plates (Nunc A/S, Roskilde, Denmark) in the presence or absence of iNOS inhibitors or S-NO-Cap.

### 2.3. NO Generation by Stimulated J774A.1 Macrophages

Nitrite concentrations as a stable final product of NO were measured by a colorimetric Griess method as described previously [18]. Briefly, equal volumes of cell culture supernatants and Griess reagent (0.5% sulfanilamide, 0.05% naphtylene-diamide dihydrochloride in 2.5% H_3_PO_4_) were mixed and incubated in room temperature for 10 min. The absorbance values were determined at 550 nm with an automated microplate reader Elx800 (BIO-TEK Instruments Inc., Winooski, VT, USA). As a standard, sodium nitrite was used. Data were expressed as *μ*M nitrite per 10^6^ cells originally plated.

### 2.4. IL-12 p40 Production

The concentration of studied cytokine in culture cell supernatants was quantitated using a sandwich ELISA. The Mouse IL-12 p40 Immunoassay Kit was purchased from R&D Systems (Minneapolis, MN, USA). ELISA was developed with horseradish peroxidase-conjugated antibody against mouse IL-12 p40 followed by tetramethylbenzidine substrate. The absorbance was read on a microplate reader Elx800 (BIO-TEK Instruments Inc., Winooski, VT, USA) at 450 nm. Recombinant murine IL-12 was used as a standard. This assay has a sensitivity of detection <4 pg/mL.

### 2.5. TNF-*α* Production

Immunoreactive TNF-*α* was estimated in cell culture supernatants by a double-antibody ELISA kit using recombinant murine TNF-*α* as a standard (R&D Systems, Minneapolis, MN, USA) following the manufacturer's protocol. The absorbance values were measured at 450 nm using the microplate reader Elx800 (BIO-TEK Instruments Inc., Winooski, VT, USA). The sensitivity of the assay was <5.1 pg/mL.

### 2.6. Determination of Cell Viability

Cell viability was determined by trypan blue dye exclusion and was assessed biochemically by measuring the cellular leakage of the cytosolic enzyme lactate dehydrogenase (LDH) using Cytotoxicity Detection Kit (Boehringer Mannheim, Mannheim, Germany). LDH activity in cell culture supernatants was measured as the amount of pyruvate consumed because of oxidation of NADH. The absorbance values were determined at 490 nm using a microplate reader Elx800 (BIO-TEK Instruments Inc., Winooski, VT, USA).

### 2.7. The Statistical Analysis

In our study each experiment was performed in quadruplicate, as the two independent experiments performed in duplicate (*n* = 4 in each group). The results are presented as the arithmetic mean and the median. The statistical differences between groups were determined by analysis of variance followed by the unpaired Student's* t*-test and the Mann-Whitney* U* test, depending on how well the results correlated with a normal distribution. Differences between the mean values were considered to be statistically significant at *P* < 0.05. The STATISTICA version 10 software (StatSoft, Cracow, Poland) was used to perform the statistical analysis.

## 3. Results

**Figure 1:**
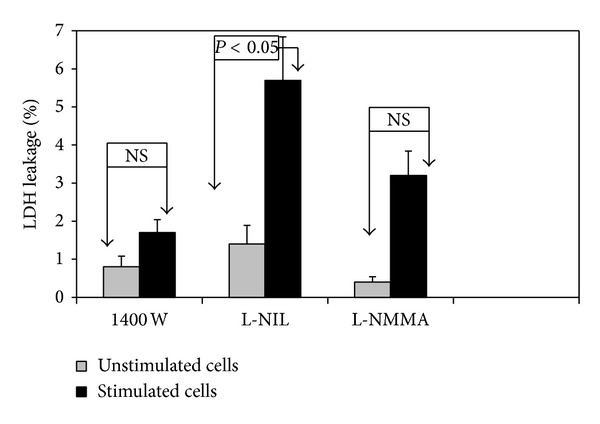
Effect of nitric oxide synthase (NOS) inhibitors (1400 W, L-NIL, and L-NMMA) on LDH release by J774A.1 macrophages. Duplicate cultures of cells (10^6^ cells/mL) were incubated at 37°C in the presence or absence (control) of stimuli: LPS (100 ng/mL) and IFN-*γ* (25 U/mL) for 18 h. Then cell culture supernatants were harvested and examined as described in Materials and Methods. All values represent means ± SD of two independent experiments performed in duplicate (*n* = 4).

The viability of the cells was greater than 92% in all performed experiments as determined by a trypan blue staining (data not shown) and LDH release (Figure 1). The accumulated nitrite in cell culture supernatants was used to estimate NO generation. The nitrite, IL-12 p40, and TNF-*α* levels were determined after 18 h of stimulation with LPS (100 ng/mL) and IFN-*γ* (25 U/mL) in the presence or absence (control) of iNOS inhibitors.

Unstimulated macrophages released a small but detectable amount of nitrite (0.82 ± 0.58 *μ*M), IL-12 p40 (171.5 ± 24.8 pg/mL), and TNF-*α* (120.5 ± 2.1 pg/mL) during 18 h incubation (data not shown). Upon activation cells produced 28.9 ± 1.7 *μ*M of nitrite, 2569 ± 393 pg/mL of IL-12 p40, and 1585 ± 358 pg/mL of TNF-*α*.

**Figure 2:**
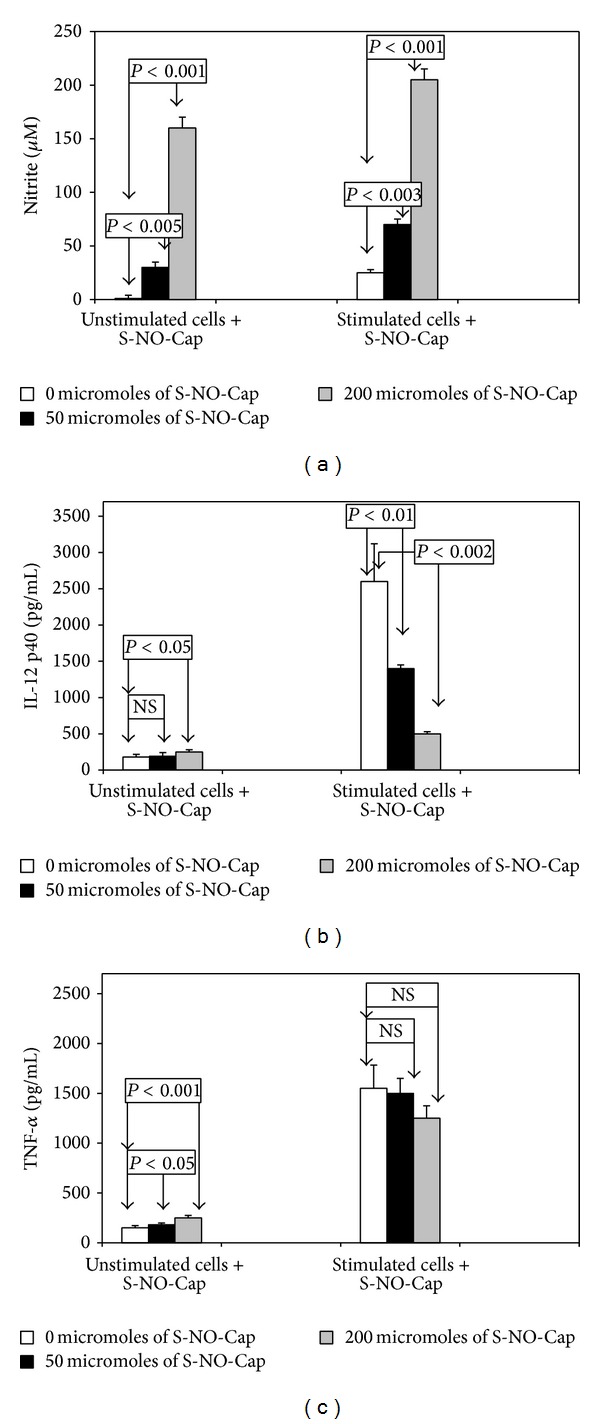
Effect of S-nitrosocaptopril (S-NO-Cap) on nitrite (a), IL-12 p40 (b), and TNF-*α* (c) production by activated J774A.1 macrophages. Duplicate cultures of cells (10^6^ cells/mL) were stimulated at 37°C with LPS (100 ng/mL) and IFN-*γ* (25 U/mL) in the presence (50 or 200 *μ*M) or absence (control) of S-NO-Cap for 18 h. Then cell culture supernatants were harvested and examined as described in Materials and Methods. All values represent means ± SD of two independent experiments performed in duplicate (*n* = 4).

In the next experiment S-NO-Cap was investigated for its involvement in cytokines secretion (Figure 2). NO generated by 200 *μ*M of S-NO-Cap significantly increased IL-12 p40 and TNF-*α* production by unstimulated cells (*P* < 0.05 and *P* < 0.001, resp.). A lower dose of S-NO-Cap (50 *μ*M) affected only TNF-*α* release (*P* < 0.05). As expected, IL-12 p40 production by stimulated cells was strongly inhibited by S-NO-Cap, but no effect on TNF-*α* release was observed.

**Figure 3:**
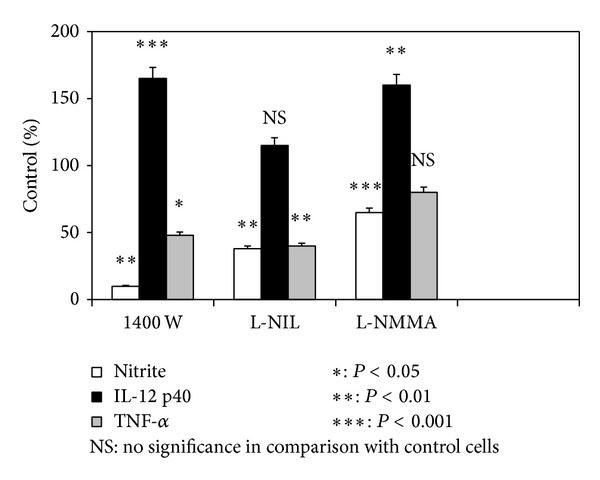
Effect of nitric oxide synthase (NOS) inhibitors on nitrite, IL-12 p40, and TNF-*α* production by activated J774A.1 macrophages. Duplicate cultures of cells (10^6^ cells/mL) were stimulated at 37°C with LPS (100 ng/mL) and IFN-*γ* (25 U/mL) in the presence or absence (control) of tested compounds: 50 *μ*M of N-[3-(aminomethyl)benzyl]acetamidine (1400 W), 50 *μ*M of L-N^G^-(1-iminoethyl)lysine (L-NIL), and 100 *μ*M of N^G^-monomethyl-L-arginine (L-NMMA). After 18 h cell culture supernatants were harvested and examined as described in Materials and Methods. Control cells produced 28.9 ± 1.7 *μ*M of nitrite, 2569 ± 393 pg/mL of IL-12 p40, and 1585 ± 358 pg/mL of TNF-*α*. All values represent means ± SD of two independent experiments performed in duplicate (*n* = 4).

The effect of iNOS inhibitors on NO, IL-12 p40, and TNF-*α* production by activated J774A.1 macrophages is shown in Figure 3. Cells were stimulated with LPS (100 ng/mL) and IFN-*γ* (25 U/mL) for 18 h in the presence or absence (control) of 1400 W (50 *μ*M), L-NIL (50 *μ*M), or L-NMMA (100 *μ*M). The most potent inhibitor of NO generation was 1400 W (7.5 ± 0.7% of control, *P* < 0.002). This compound also markedly increased IL-12 p40 secretion (163.8 ± 12.1% of control, *P* < 0.001) and decreased TNF-*α* release (44.8 ± 0.7% of control, *P* < 0.02). L-NIL suppressed both NO and TNF-*α* production, but it did not change IL-12 p40 synthesis. The effect of L-NMMA on NO generation was weaker than other inhibitors. Moreover, it decreased TNF-*α* secretion slightly (86.8 ± 5.9% of control) but not significantly.

## 4. Discussion 

### 4.1. Effect of an Exogenous NO

In the present study an exogenous NO (generated by S-NO-Cap) affected cytokine release by macrophages. It markedly suppressed IL-12 production by LPS/IFN-*γ* induced J774A.1 macrophages, which is in agreement with Huang et al.'s report [5], in which NO generated by S-nitroso-N-acetyl-penicillamine inhibits the production of IL-12 protein and IL-12 p40 mRNA expression. NO did not change TNF-*α* synthesis in our model of stimulated cells. Earlier it was reported that NO might both enhance [19] and attenuate TNF-*α* production [20] in the different cell lines. Moreover, we showed that treatment of unstimulated J774A.1 cells with NO donor resulted in the increase of the basal levels of IL-12 and TNF-*α*. NO could control the Th1 cell development through a feedback mechanism that suppressed IL-12 synthesis [5]. Taken together, our results confirmed the regulatory effect of NO on cytokine release.

### 4.2. Effect of NOS Inhibitors

Overexpression of individual NOS isoforms plays a role in a wide range of disorders, including septic shock, arthritis, asthma, diabetes, ischemia-reperfusion injury, and the various neurodegenerative diseases [8, 9, 21]. Knowledge of the physical locations and functions of the various isoforms of NOS is necessary when considering manipulations of their expression. Unfortunately, nonselective NOS inhibitors also inactivate the constitutive isoforms and their administration might cause a marked and sustained increase in blood pressure.

NO production by J774A.1 macrophages has been mainly attributed to inducible NO synthase (iNOS) activity, which is induced by inflammatory cytokines or bacterial products, such as LPS. The iNOS has become a reliable biomarker for fully activated macrophages, whereas information regarding roles for endothelial (eNOS) and neuronal (nNOS) nitric oxide synthase during macrophage activation has been limited [22].

1400 W, a nontoxic for the cells novel NOS inhibitor, is the most selective inhibitor of iNOS isoform described to date [11, 12, 23]. It is greater than 5000-fold and 200-fold more potent against purified human iNOS than eNOS and nNOS, respectively [12]. Treatment with 1400 W at effective doses on iNOS did not have effect on basal systemic blood pressure or on exacerbating early effect of LPS on vascular leakage, whereas treatment with nonselective inhibitor N-nitro-L-arginine methyl ester (L-NAME) caused a significant increase in blood pressure and exacerbation of early vascular leak [24]. A beneficial action of 1400 W on the colonic injury using an experimental model of colitis in rats has been reported [25, 26]. Treatment with 1400 W reduced neutrophil infiltration, edema formation, and acute inflammatory damage in induced acute colitis [25]. It was a potent inhibitor (150-fold more potent than L-NMMA) of LPS-provoked colonic vascular injury in rat model [12, 24]. L-NIL, a relatively selective inhibitor of iNOS, suppressed the increase in the plasma nitrite levels and joint inflammation associated with adjuvant-induced arthritis. Inhibition was observed at doses, which did not appear to inhibit eNOS, as determined by a lack of effect on systemic blood pressure [27]. L-NIL was considerably more potent than L-NMMA in suppressing nitrite accumulation by intact macrophages [28]. Ruetten et al. suggest that selective inhibitors of iNOS activity might attenuate the liver and pancreatic dysfunction caused by endotoxemia in rats [29].

Considerable evidences suggest that 1400 W exerts an anti-inflammatory activity and it is related to its ability of suppressing NO generation. Additionally, this inhibitor could modify the host immune response via the affectation other mediators production. Indeed, we showed that 1400 W decreased TNF-*α* release. TNF-*α*, a principle proinflammatory cytokine, is responsible for a diverse range of signaling events within cells, leading to necrosis or apoptosis. It produces fever, inflammation, tissue destruction, and (in some cases) shock and death [30]. Moreover, 1400 W potently increased a secretion of p40 subunit of IL-12. Interestingly, a p40 homodimer may function as an IL-12 antagonist by binding to the IL-12 receptor, but not by mediating a biologic response [31]. The potency of inhibition of NO production is 1400 W > L-NIL > L-NMMA, but neither IL-12 p40 nor TNF-*α* production follows this pattern. It is possible because IL-12 production could be perturbed by the excess of endogenous NO. For example, Suzuki et al. [13] suggest that IL-12 production in stimulated J774A.1 cells inhibited by 1400 W could be increased by limiting endogenous NO production.

The potency and selectivity of 1400 W as an inhibitor of iNOS and cytokine release modifier are encouraging for therapeutic use. However, the designing of appropriate strategies for an intervention requires further studies* in vitro* and* in vivo*.

## References

[1] Guzik TJ, Korbut R, Adamek-Guzik T (2003). Nitric oxide and superoxide in inflammation and immune regulation. *Journal of Physiology and Pharmacology*.

[2] Verma N, Chakrabarti R, Das RH, Gautam HK (2012). Anti-inflammatory effects of shea butter through inhibition of iNOS, COX-2, and cytokines via the Nf-Kb pathway in Lps-activated J774 macrophage cells. *Journal of Complementary and Integrative Medicine*.

[3] Lamont AG, Adorini L (1996). IL-12: a key cytokine in immune regulation. *Immunology Today*.

[4] Brahmachari S, Pahan K (2009). Suppression of regulatory T cells by IL-12p40 homodimer via nitric oxide. *The Journal of Immunology*.

[5] Huang F-P, Niedbala W, Wei X-Q (1998). Nitric oxide regulates Th1 cell development through the inhibition of IL-12 synthesis by macrophages. *European Journal of Immunology*.

[6] Ma X, Sun J, Papasavvas E (2000). Inhibition of IL-12 production in human monocyte-derived macrophages by TNF. *The Journal of Immunology*.

[7] Ignarro LJ (2002). Nitric oxide as a unique signaling molecule in the vascular system: a historical overview. *Journal of Physiology and Pharmacology*.

[8] Prado CM, Martins MA, Tiberio IFLC (2011). Nitric oxide in asthma physiopathology. *ISRN Allergy*.

[9] Zamora R, Vodovotz Y, Billiar TR (2000). Inducible nitric oxide synthase and inflammatory diseases. *Molecular Medicine*.

[10] Ortega MG, Saragusti AC, Cabrera JL, Chiabrando GA (2010). Quercetin tetraacetyl derivative inhibits LPS-induced nitric oxide synthase (iNOS) expression in J774A.1 cells. *Archives of Biochemistry and Biophysics*.

[11] Boer R, Ulrich W-R, Klein T, Mirau B, Haas S, Baur I (2000). The inhibitory potency and selectivity of arginine substrate site nitric-oxide synthase inhibitors is solely determined by their affinity toward the different isoenzymes. *Molecular Pharmacology*.

[12] Garvey EP, Oplinger JA, Furfine ES (1997). 1400 W is a slow, tight binding, and highly selective inhibitor of inducible nitric-oxide synthase *in vitro* and *in vivo*. *The Journal of Biological Chemistry*.

[13] Suzuki C, Aoki-Yoshida A, Kimoto-Nira H, Kobayashi M, Sasaki K, Mizumachi K (2014). Effects of strains of *Lactococcus lactis* on the production of nitric oxide and cytokines in murine macrophages. *Inflammation*.

[14] Stuehr DJ, Marletta MA (1987). Synthesis of nitrite and nitrate in murine macrophage cell lines. *Cancer Research*.

[15] Nosaka C, Kunimoto S, Atsumi S, Takeuchi T (1999). Inhibition of nitric oxide synthase induction by 15-deoxyspergualin in a cultured macrophage cell line, J744A.1 activated with IFN-*γ* and LPS. *Journal of Antibiotics*.

[16] Kizaki T, Suzuki K, Hitomi Y (2001). Negative regulation of LPS-stimulated expression of inducible nitric oxide synthase by Ap-1 in macrophage cell line J774A.1. *Biochemical and Biophysical Research Communications*.

[17] Banjanac M, Munić Kos V, Nujić K (2012). Anti-inflammatory mechanism of action of azithromycin in LPS-stimulated J774A.1 cells. *Pharmacological Research*.

[18] Green LC, Wagner DA, Glogowski J, Skipper PL, Wishnok JS, Tannenbaum SR (1982). Analysis of nitrate, nitrite, and [^15^N]nitrate in biological fluids. *Analytical Biochemistry*.

[19] Huang F-P, Niedbala W, Wei X-Q (1998). Nitric oxide regulates Th1 cell development through the inhibition of IL-12 synthesis by macrophages. *European Journal of Immunology*.

[20] Eigler A, Moeller J, Endres S (1995). Exogenous and endogenous nitric oxide attenuates tumor necrosis factor synthesis in the murine macrophage cell line RAW 264.7. *The Journal of Immunology*.

[21] Egilmez NK, Harden JL, Virtuoso LP, Schwendener RA, Kilinc MO (2011). Nitric oxide short-circuits interleukin-12-mediated tumor regression. *Cancer Immunology, Immunotherapy*.

[22] Huang Z, Hoffmann FW, Fay JD (2012). Stimulation of unprimed macrophages with immune complexes triggers a low output of nitric oxide by calcium-dependent neuronal nitric-oxide synthase. *The Journal of Biological Chemistry*.

[23] Ghosh DK, Misukonis MA, Reich C, Pisetsky DS, Weinberg JB (2001). Host response to infection: The role of CpG DNA in induction of cyclooxygenase 2 and nitric oxide synthase 2 in murine macrophages. *Infection and Immunity*.

[24] László F, Whittle BJR (1997). Actions of isoform-selective and non-selective nitric oxide synthase inhibitors on endotoxin-induced vascular leakage in rat colon. *European Journal of Pharmacology*.

[25] Kankuri E, Vaali K, Knowles RG (2001). Suppression of acute experimental colitis by a highly selective inducible nitric-oxide synthase inhibitor, N-[3-(aminomethyl)benzyl]acetamidine. *Journal of Pharmacology and Experimental Therapeutics*.

[26] Menchén LA, Colón AL, Moro MA (2001). N-(3-(Aminomethyl)benzyl)acetamidine, an inducible nitric oxide synthase inhibitor, decreases colonic inflammation induced by trinitrobenzene sulphonic acid in rats. *Life Sciences*.

[27] Connor JR, Manning PT, Settle SL (1995). Suppression of adjuvant-induced arthritis by selective inhibition of inducible nitric oxide synthase. *European Journal of Pharmacology*.

[28] Stenger S, Thüring H, Röllinghoff M, Manning P, Bogdan C (1995). L-N^G^-(1-Iminoethyl)-lysine potently inhibits inducible nitric oxide synthase and is superior to N^G^-monomethyl-arginine *in vitro* and *in vivo*. *European Journal of Pharmacology*.

[29] Ruetten H, Southan GJ, Abate A, Thiemermann C (1996). Attenuation of endotoxin-induced multiple organ dysfunction by 1-amino-2-hydroxy-guanidine, a potent inhibitor of inducible nitric oxide synthase. *British Journal of Pharmacology*.

[30] Dinarello CA (2000). Proinflammatory cytokines. *Chest*.

[31] Stern AS, Magram J, Presky DH (1996). Interleukin-12 an integral cytokine in the immune response. *Life Sciences*.

